# Multiomics analyses reveals *Anaplasma phagocytophilum* Ats-1 induces anti-apoptosis and energy metabolism by upregulating the respiratory chain-mPTP axis in eukaryotic mitochondria

**DOI:** 10.1186/s12866-022-02668-x

**Published:** 2022-11-11

**Authors:** Ruirui Li, Zhongchen Ma, Wei Zheng, Zhen Wang, Jihai Yi, Yangyang Xiao, Yong Wang, Chuangfu Chen

**Affiliations:** 1 International Research Center for Animal Health Breeding, College of Animal Science and Technology, Shihezi University, Shihezi, China; 2 Collaborative Innovation Center for Prevention and Control of High Incidence Zoonotic Infectious Diseases in Western China, College of Animal Science and Technology, Shihezi University, Shihezi, China

**Keywords:** *Anaplasma phagocytophilum*, Mitochondria, Ats-1, iTRAQ, PRM, Targeted metabonomics

## Abstract

**Background:**

Anaplasma translocated substrate 1 (Ats-1) is an effector of type 4 secretory systems (T4SS) and the main virulence factor of *Anaplasma phagocytophilum*. Ats-1 is involved in the regulation of host cell biological processes, but the specific molecular mechanism of its action is unclear.

**Results:**

In this study, we identified Ats-1 as involved in mitochondrial respiratory regulation of HEK293T cells by multi-omics analysis. After intracellular expression of Ats-1, adenosine triphosphate levels and the proliferation of HEK293T cells were both up-regulated, while HEK293T cells apoptosis was inhibited. Ats-1 targeted translocation to the mitochondria where it up-regulated the expression of NDUFB5, NDUFB3, NDUFS7, COX6C, and SLC25A5, thereby enhancing energy production and inhibiting HEK293T cells apoptosis while enhancing HEK293T cells proliferation, and ultimately facilitating *Anaplasma phagocytophilum* replication in HEK293T cells.

**Conclusions:**

This study demonstrated that *Anaplasma phagocytophilum* Ats-1 induces anti-apoptosis and energy metabolism by upregulating the respiratory chain-mPTP axis in eukaryotic mitochondria. These results provide a better understanding of the pathogenic mechanism of *Anaplasma phagocytophilum* within host cells.

**Supplementary Information:**

The online version contains supplementary material available at 10.1186/s12866-022-02668-x.

## Introduction

Human granulocytic anaplasmosis is a tick-borne zoonotic disease caused by *Anaplasma phagocytophilum*. *A. phagocytophilum* is an intracellular parasite that can survive long periods within host cells including neutrophils, monocytes, macrophages, epithelial cells, and others[1, 2]. After *A. phagocytophilum* infection occurs in animals and humans, symptoms such as leukopenia, thrombocytopenia, and increased serum aminotransferase (liver enzyme) activity are often present[1–5]. The disease is commonly present in the northeastern USA, northern Europe, and southeastern Asia [6–9]. The specific molecular mechanism of *A. phagocytophilum* infection of host cells is unclear, seriously hindering research into new treatment and preventive measures.T4SS transfers proteins or nucleoprotein complexes from bacteria to eukaryotic cells through eukaryotic cell membranes [10]. Three *A. phagocytophilum* T4SS effectors have been experimentally identified including AnkA, APH-0455, and Ats-1 [11–13]. Among these, Ats-1 is the first bacterial protein known to pass through five layers of membranes. When Ats-1 is in the mitochondria, it can block the loss of mitochondrial membrane potential and inhibit host cell apoptosis that is induced by etoposide [12]. Further, Ats-1 targeting *A. phagocytophilum* inclusion bodies binds to Beclin-1 and induces autophagy [14]. However, the regulatory mechanisms of Ats-1 remain unclear and require further investigation.

Proteomics and metabonomics have been widely used in recent years to study the mechanisms underlying various diseases like tuberculosis [15], liver fluke infection [16, 17], and diabetes [18]. In addition, isobaric tags used in the relative and absolute quantification (iTRAQ) [19–21]of proteins can be used to evaluate differentially expressed proteins under different biological conditions and has attracted widespread attention. However, iTRAQ analysis cannot achieve the identification of specific core proteins [22]. Parallel reaction monitoring (PRM) is an ion monitoring technique that is based on high resolution and high precision mass spectrometry that are used in the relative or absolute quantification of target proteins and peptides [23]. PRM can consequently be used to validate iTRAQ results. In addition, targeted metabolomics analyzes several target compounds or metabolites involved in certain pathways, while using standards to construct detection methods with high specificity, high sensitivity, and good repeatability.

In this study, proteomics (iTRAQ) was used to investigate the proteome changes in HEK293T cells after Ats-1 expression, and PRM, targeted metabolomics, and other experiments were used to verify the regulation of Ats-1 in HEK293T cells, which laid a foundation for the in-depth understanding of the molecular pathogenesis of *A. phagocytophilum*.

## Materials and methods

### Bioinformatics analyses

The Signalp 5.0 (http://www.cbs.dtu.dk/services/SignalP/) and TargetP 2.0 (http://www.cbs.dtu.dk/services/TargetP/) servers were used to predict signal peptides and mitochondrial transfer peptides of the Ats-1 protein, respectively. The secondary structure of the Ats-1 protein was predicted and analyzed with the SOPM software program (https://npsa-prabi.ibcp.fr/cgi-bin/npsa_automat.pl?page=/NPSA/npsa_sopma.html). The tertiary structure of the Ats-1 protein was predicted and analyzed with the Swiss-Model software program (http://swissmodel.expasy.org/). The CNLS Mapper software program (http://nls-mapper.iab.keio.ac.jp/cgi-bin/NLS_Mapper_form.cgi) was used to predict the nuclear localization signal (NLS) and the NetNES 1.1 server (http://www.cbs.dtu.dk/services/NetNES/) was used to analyze the nuclear export signal (NES).

### Cells

HEK293T cells were provided by the Cell Resource Center at the Institute of Basic Medical Sciences of the Chinese Academy of Medical Sciences/Peking Union Medical College (Beijing, China), and were cultured in DMEM (Solarbio, Beijing, China) medium containing 10% fetal bovine serum (FBS) (Gibco, Brooklyn, NY, USA) in an incubator with a 5% CO_2_ atmosphere.

### Construction of a eukaryotic expression vector and establishment of a transfection model

The Ats-1 gene was synthesized according to the sequence deposited in Genbank (Accession: FJ210653) and was cloned into the pcDNA3.1 vector with a His tag by Sangon Biotech (Shanghai, China).The pcDNA3.1-Ats-1 plasmid was extracted with a large-scale plasmid extraction kit (Tiangen, Beijing, China) according to the manufacturer’s instructions. HEK293T cells were cultured until reaching cell densities of 80–90%. The cells were divided into three groups including group A: pcDNA3.1-Ats-1 plasmid transfection into cells, group P: transfection with the pcDNA3.1 empty vector, and group H: the no transfection control group. The Lipofectamine plasmid 3000 Transfection Reagent (Invitrogen, Carlsbad, USA) was used for transfection. After 48 h, the supernatant was discarded and native lysis buffer (Solarbio, Beijing, China) along with protease inhibitors (Solarbio, Beijing, China) were added, followed by total protein extraction and storage at − 80 °C.

### Verification of transfection by Western blot analysis

For western blotting (WB), the proteins were resolved on SDS–polyacrylamide gel electrophoresis (SDS–PAGE) gels followed by standard WB. Rabbit anti-6×His tag antibody (Abcam, Cambridge, MA, USA) (1:2,000) as the primary antibody. Goat anti-rabbit IgG H&L (Abcam, Cambridge, MA, USA) (1:4,000) was used as the secondary antibody and a SuperSignal West Femto Trial Kit (Thermo Fisher Scientific, Waltham, MA, USA) was used for color development. β-Actin protein was used as the control, using mouse anti-β-actin monoclonal antibody (Sino Biological, Beijing, China) (1:2,000) and goat anti-mouse IgG H&L (Abcam, Cambridge, MA, USA) (1:4,000).

### iTRAQ, PRM, and targeted metabolomics

The total cellular proteins from the three groups of cells were extracted, quantified, detected, digested, desalted, and labeled for iTRAQ according to methods described by Ross et al. [24]. An EASY-nLCTM 1,200 na upgrade UHPLC system with a Q ExactiveTM HF-X mass spectrometer were used to generate mass spectra for detecting the original data. The Proteome Discoverer 2.2 software program was used to search the database (homo_sapiens_uniprot_2019.01.18.fasta), followed by quantification of peptides and proteins. iTRAQ LC-MS/MS proteome analysis was performed by Novogene Co., Ltd. (Beijing, China). Additional quantification (PRM) of eight differentially expressed proteins was also conducted by Novogene Co., Ltd. (Beijing, China), along with targeted metabolomics to quantify and analyze carbon-related compounds that were present in all three sample groups. Three samples in each group were used for iTRAQ, PRM, and targeted metabolomic analysis.

### Quantitative real-time PCR (qRT-PCR) detection

The total RNAs from the three groups of transfected cells were extracted with an Ultrapure RNA Kit (CWBIO, Beijing, China). RNA purity and concentration were determined with a nucleic acid detector (Nanodrop ND-2000, Wilmington, DE, USA). RNA was then reverse transcribed into cDNA using a HiFiScript cDNA Synthesis Kit (CWBIO, Beijing, China). The UltraSYBR mixture (High ROX) (CWBIO, Beijing, China) was used for quantitative detection. Glyceraldehyde 3-phosphate dehydrogenase (GAPDH) was used as the internal reference (primer sequence shown in Supplementary Table 1). qRT-PCR was conducted using a QuantStudio™ 5 Real-Time PCR System (Thermo Fisher Scientific, Waltham, MA, USA).

### Adenosine triphosphate (ATP) level detection

Cell lysate protein concentration was detected using the Pierce BCA Protein Assay Kit (Thermo Fisher Scientific, Waltham, MA, USA), followed by measurement of absorbance at 570 nm with a multi-function enzyme labeling instrument (SUNRISE, Tecan Austria Gmbh, Grodig, Austria). Protein concentrations were then calculated according to a standard curve. The ATP concentrations were also measured for the three groups of cells after transfection using an Enhanced ATP Assay Kit (Beyotime, Shanghai, China) following the manufacturer’s instructions. Chemiluminescence was detected using a multi-functional detector (Synergy 2 Biotek USA).

### CCK-8 detection of cell proliferation

After 48 h post-transfection, the three groups of cells were inoculated in a 96 well culture plate at 2 × 10^3^ cells per well and then cultured at 37 °C for 2 h in an incubator with a 5% CO_2_ atmosphere. Cell proliferation was detected with a Cell Counting Kit-8 (Dojindo Laboratories, Shanghai, China). The absorbance value (A_450_) of each well was then determined with a multi-function enzyme labeling instrument (SUNRISE, Tecan Austria Gmbh, Grodig, Austria).

### Detection of HEK293T cells apoptosis by flow cytometry

HEK293T cells in logarithmic growth phase were inoculated into six-well cell culture plates at 2 × 10^4^ cells/well. Cells were divided into three groups for transfection, and three duplicate wells were set up in each group and cultured for 48 h at 37 °C in a 5% CO_2_ incubator. Cell apoptosis was evaluated with the Annexin V-FITC/PI Apoptosis Detection Kit (YEASEN, Shanghai, China) and flow cytometry (BD FACSAria™ III, BD Biosciences, Franklin Lakes, NJ, USA).

### Data analysis

The data reported here represent the results from three independent experiments, with values as means ± SD. The GraphPad Prism software program was used to construct figures and the SPSS software program was used for statistical analyses. NSK tests and one-way ANOVA tests were used to compare means among different groups of data, wherein *: *p* < 0.05, *: *p* < 0.01, ***: *p* < 0.001, and ****: *p* < 0.0001 were considered statistically significant.

## Results

### Ats-1 Bioinformatics analyses

To more comprehensively understand the function of the Ats-1 protein, it was analyzed using several bioinformatics software tools. The results showed that Ats-1 had Sec and Tat signal peptides (Supplementary Fig. 1 A) in addition to mitochondrial transfer signals (Supplementary Fig. 1 B). Protein secondary structures indicated that α-helices of Ats-1 were the most abundant structures, followed by β-folding and β-turning angles, with each accounting for 39.89%, 8.78%, and 6.38%, respectively (Supplementary Fig. 1 C). The tertiary structure prediction of Ats-1 (Supplementary Fig. 1 D) revealed a protein coverage rate of 26.98%. The CNLS Mapper software program and the NetNES 1.1 server prediction results showed that Ats-1 had no NLS or NES (Supplementary Fig. 1 E, 1 F). Predictive analysis further revealed that Ats-1 may transfer to the endoplasmic reticulum and across the bilayer lipid membrane of mitochondria, but not into nuclei.

###  Successful expression of Ats-1 in HEK293T cells

**Fig. 1:**
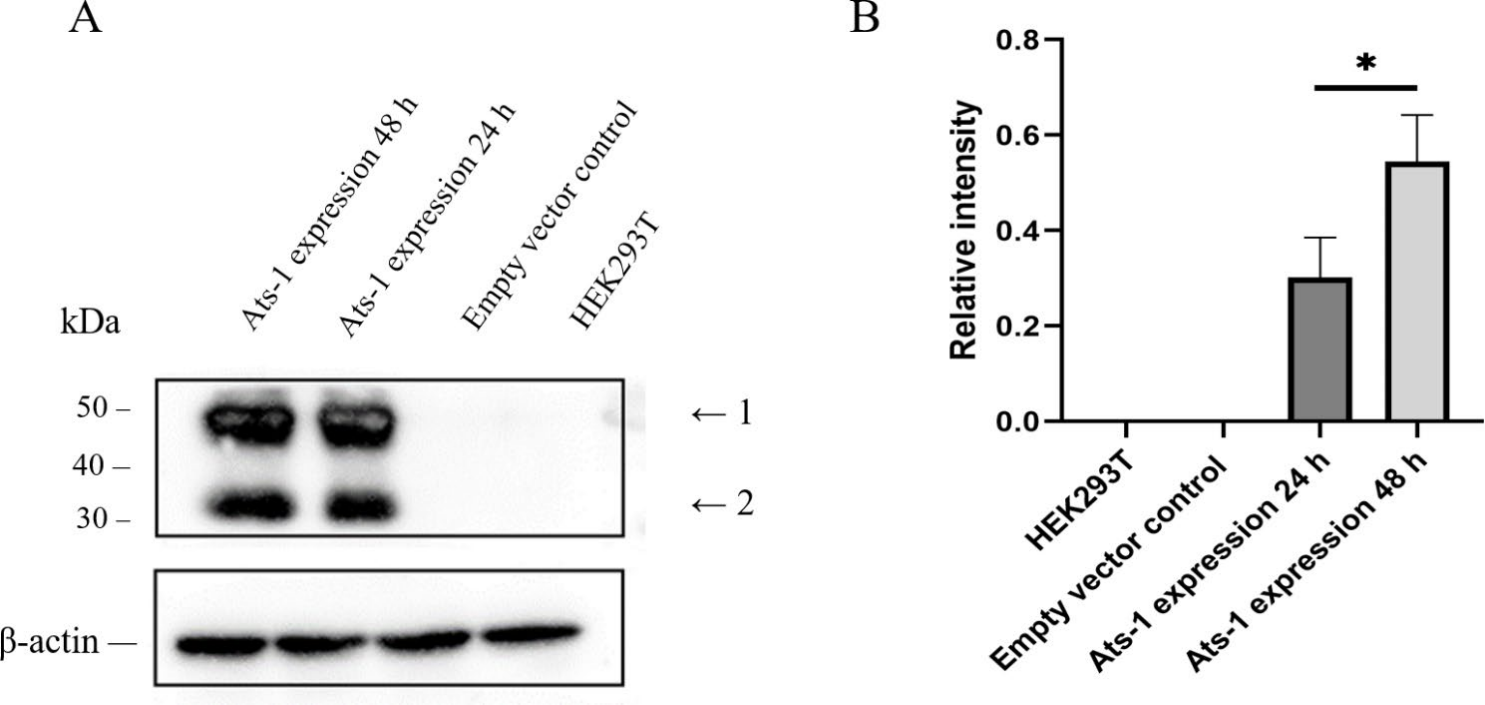
(A) Ats-1 was expressed in HEK293T cells for 24 and 48 h, then transfected with empty vector and untransfected HEK293T cells for Western blot analysis. Each band in the figure represents the results of three independent experiments. Arrows 1 and 2 indicate full-length Ats-1 and cleaved Ats-1 proteins, respectively [14]. Molecular mass markers are shown on the left. The original diagram is shown in Supplementary Fig. 2. (B) Western blot results that were semi-quantitatively analyzed using the ImageJ software program. All data are shown as means ± SD from three independent tests. *: *p* < 0.05.

After transfection of HEK293T cells with pcDNA3.1-Ats-1 for 24 and 48 h, total cell proteins were collected and analyzed by Western blot. Two Ats-1 protein forms have been identified in cells including the full-length 48 kDa protein and the 35 kDa truncated Ats-1 protein [12]. Clear target bands were observed at 48 kDa and 35 kDa, indicating obvious expression of Ats-1 proteins (Fig. 1 A). Semi-quantitative analysis with the ImageJ software program revealed that target protein expression at 48 h after transfection was significantly higher than at 24 h (*p* < 0.05) (Fig. 1 B). Thus, Ats-1 proteins were successfully expressed in HEK293T cells.

### Proteomic analysis of HEK293T cells after Ats-1 expression

**Fig. 2:**
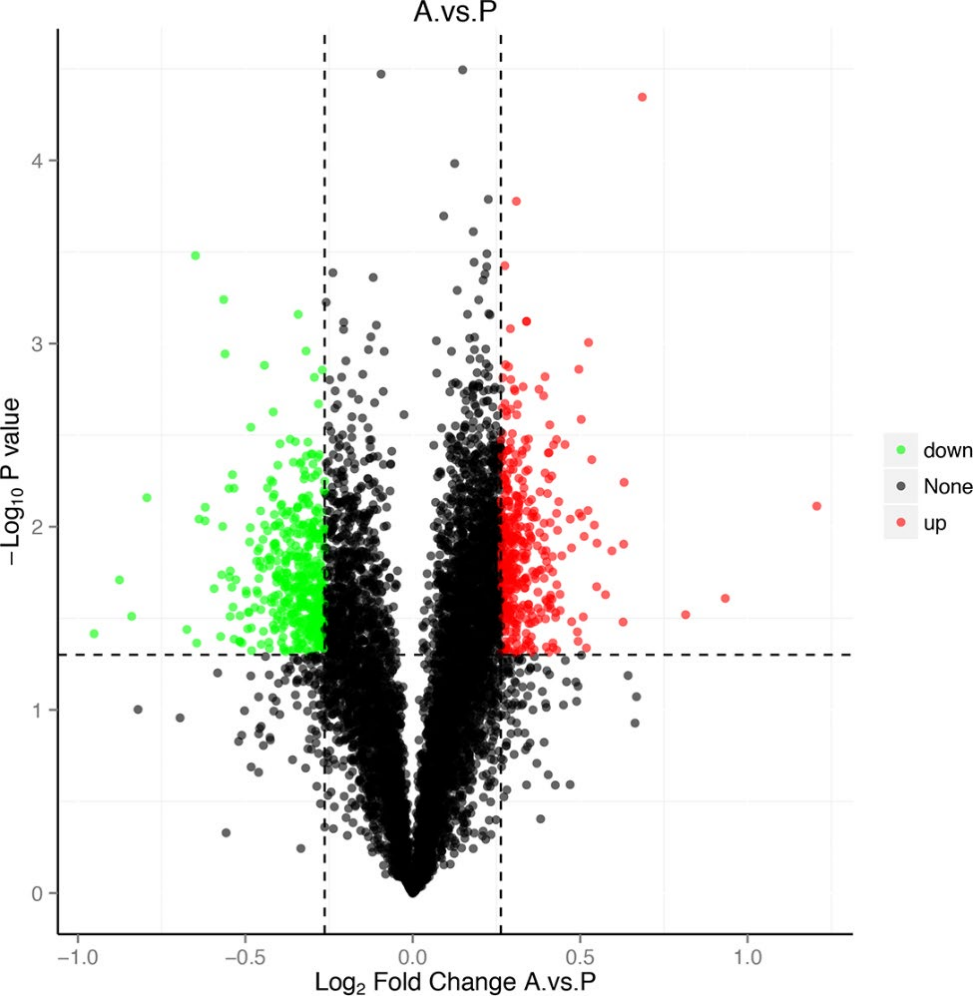
Volcano plots showing differentially expressed proteins detected in Ats-1-transfected HEK293T cells compared to control HEK293T cells (A. vs. P). The x‑axis represents log2 (fold change) values while the y‑axis represents − log10(p) values.

To fully understand proteome changes in host cells after Ats-1 expression, HEK293T cells were transfected with empty pcDNA3.1 vector (group P) and pcDNA3.1-Ats-1 recombinant vector (group A). Cells were then collected 48 h later for LC-MS based proteomics analyses. The mass spectrometry results were compared against a human database (homo_sapiens_uniprot_2019.01.18.fasta) to identify proteins. The expression levels of 862 proteins were identified as significantly different between the two groups (*p* < 0.05, fold change > 2). A total of 407 proteins were up-regulated in group A, while 455 proteins were down-regulated (Fig. 2).

**Fig. 3:**
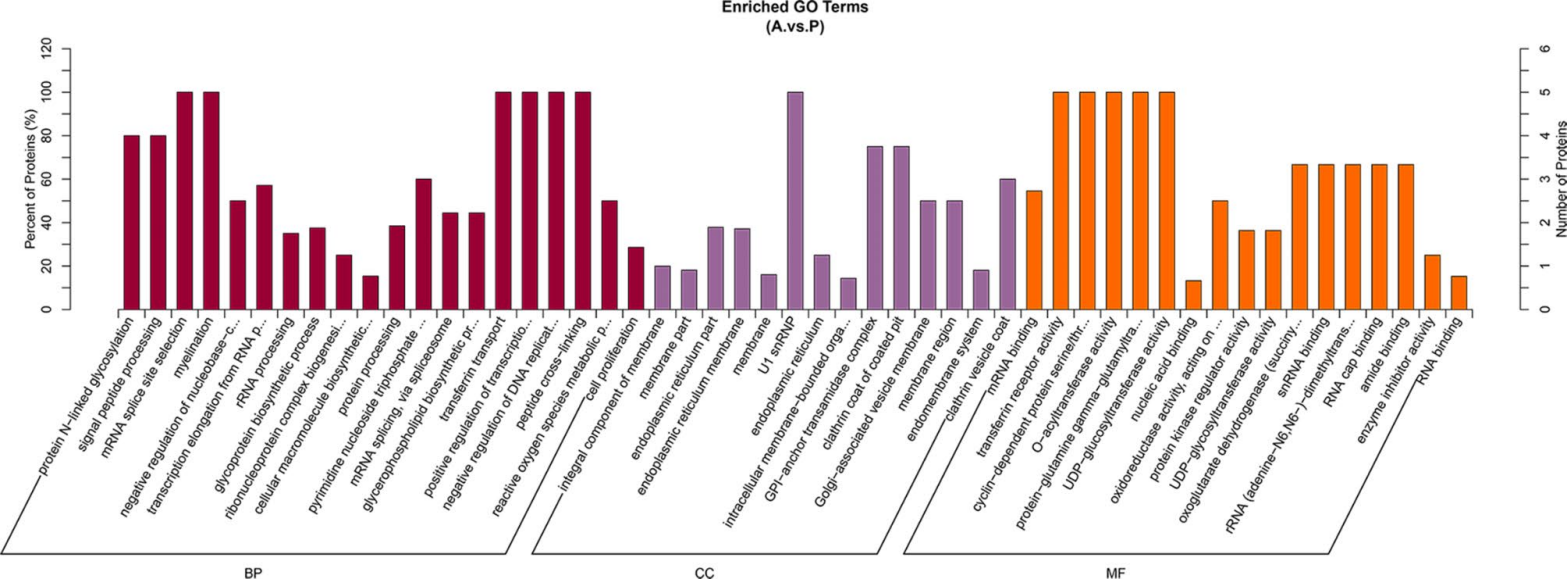
Gene ontology (GO) analysis of total proteins based on the GO categories of cellular components (CC), biological processes (BP), and molecular functions (MF).

**Fig. 4:**
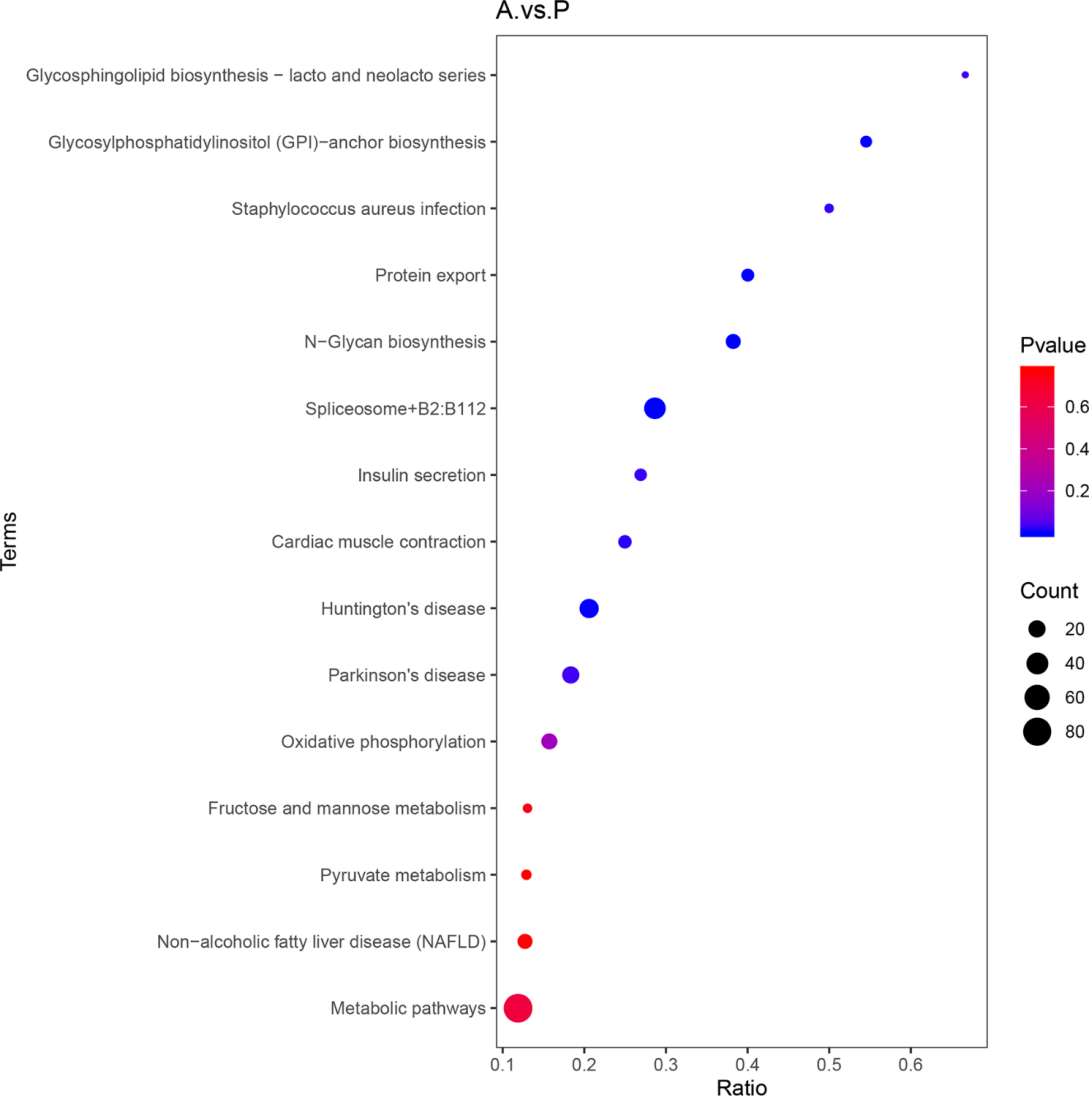
KEGG enrichment analyses of differentially abundant proteins (Fisher’s exact test, *p* < 0.05).

To determine the biological processes and pathways involved in Ats-1 expression, significantly differentially expressed proteins were compared against functional databases including the Gene Ontology (GO) database and the Kyoto Encyclopedia of Genetics and Genomes (KEGG) [25, 26]database. GO analysis is divided into three functional categories: biological processes (BP), cellular components (CC), and analytical functions (MF). Among the annotations of differentially expressed proteins into the three functional categories, reactive oxygen species metabolic processes were identified in BP, while oxidoreductase activity via acting on the aldehyde or oxo groups of donors and oxoglutarate dehydrogenases (succinyl-transferring) were identified in the MF category (Fig. 3). These three BP are all related to mitochondrial function. In both the GO and KEGG analysis, oxidative phosphorylation and pyruvate metabolism were both highly enriched (Fig. 4), which are also related to mitochondrial function. Thus, differentially expressed proteins after Ats-1 expression in HEK293T cells were particularly associated with mitochondrial-related pathways.

### PRM validation of mitochondrial respiratory chain-related protein expression

**Fig. 5:**
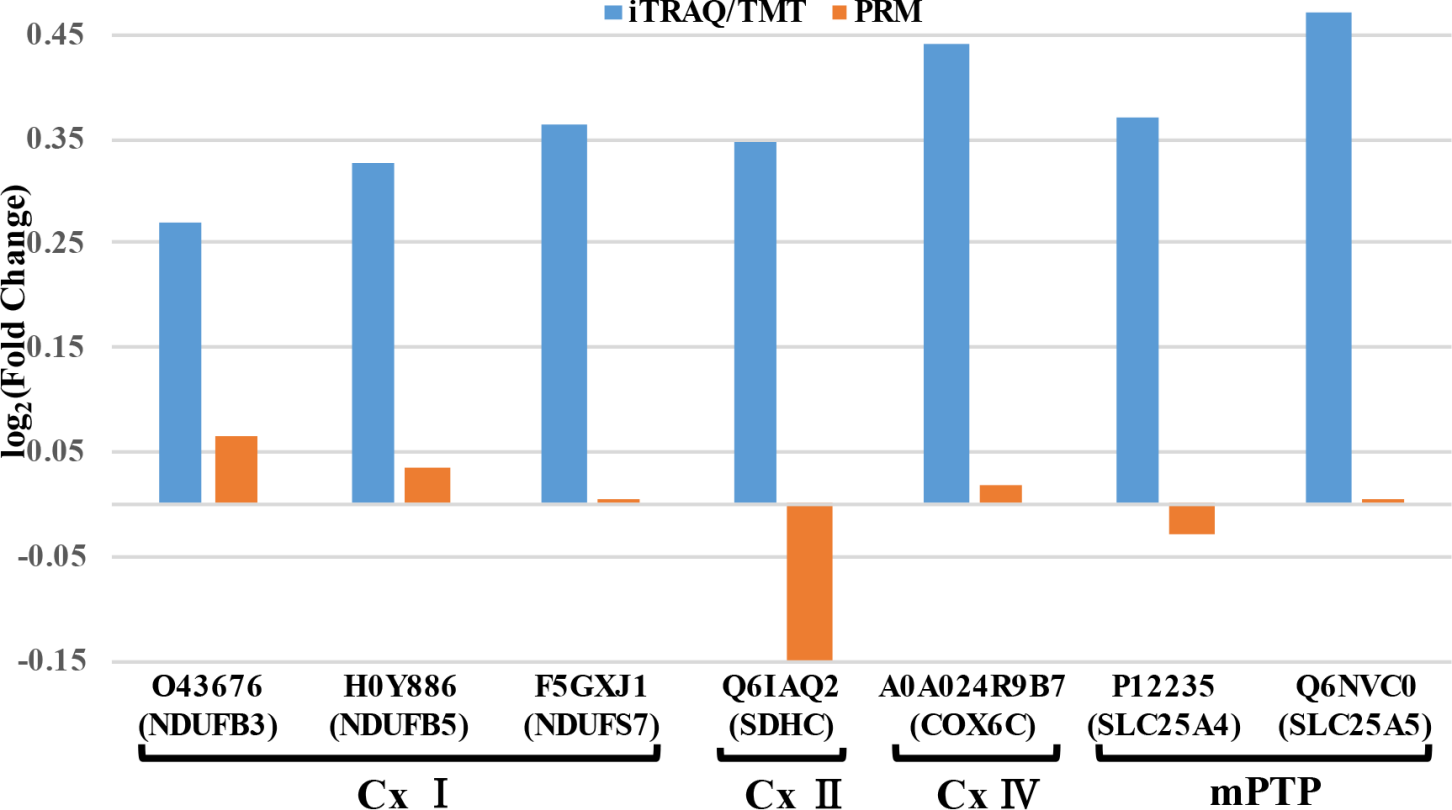
Proteins identified by iTRAQ analysis (A. vs. P groups) and verified by PRM analysis. The iTRAQ data were validated using seven proteins.

Niu [12] also observed that Ats-1 contained a signal peptide targeting mitochondria, and that after the recombinant vector PGADT7-Ats-1 was transfected into yeast, the Ats-1 protein was observed in mitochondria. The differential proteins identified in the experiments of this study were also enriched with mitochondrial functions based on GO and KEGG analyses. Seven of 862 differentially expressed proteins related to mitochondrial biological function (Table 1) were selected for PRM analysis alongside iTRAQ analysis (Fig. 5). After Ats-1 expression, the expression levels of five proteins (NDUFB3, NDUFB5, NDUFS7, COX6C, and SLC25A5) based on iTRAQ were the same as identified by PRM (i.e., they were all up-regulated).

**Table 1:** Seven differentially expressed proteins related to the mitochondrial respiratory chain and mPTP selected in iTRAQ.

Protein		Gene	Description	Fold change	*P*-value
Q6NVC0		SLC25A5	SLC25A5 protein (fragment)	1.38	0.021
P12235		SLC25A4	ADP/ATP translocase 1	1.26	0.008
H0Y886		NDUFB5	NADH dehydrogenase [ubiquinone] 1 beta subcomplex subunit 5, mitochondrial (fragment)	1.24	0.013
F5GXJ1		NDUFS7	NADH dehydrogenase [ubiquinone] iron-sulfur protein 7, mitochondrial	1.23	0.024
A0A024R9B7		COX6C	Cytochrome c oxidase subunit Vic, isoform CRA_a	1.34	0.009
O43676		NDUFB3	NADH dehydrogenase [ubiquinone] 1 beta subcomplex subunit 3	1.26	0.019
Q6IAQ2		SDHC	SDHC protein	1.47	0.002

### Ats-1 Up-regulates the expression of 80% of central carbon pathway-related metabolites

**Fig. 6:**
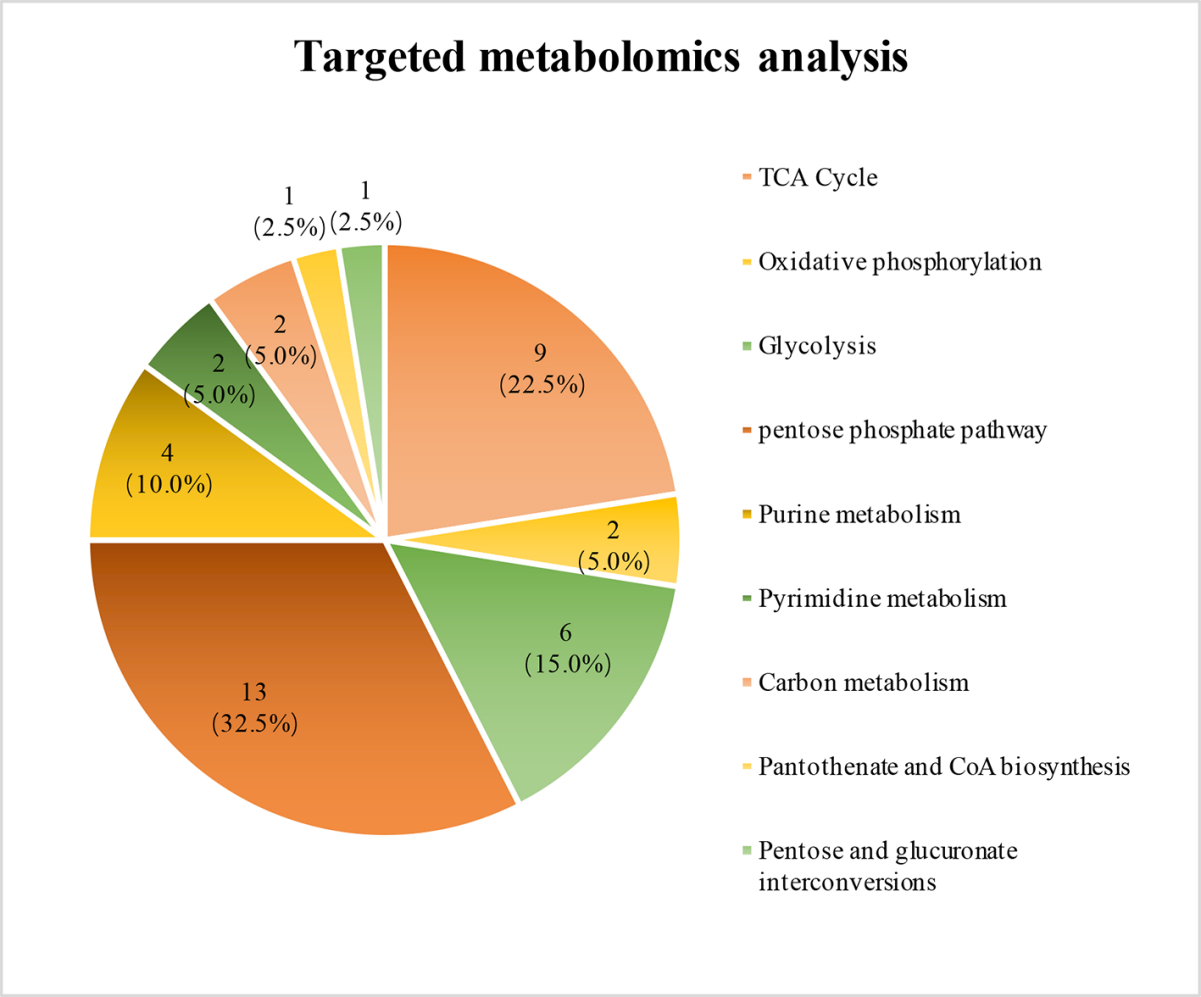
Targeted metabolic analysis showing coverage of the detected substances relative to the central carbon metabolite.

**Fig. 7:**
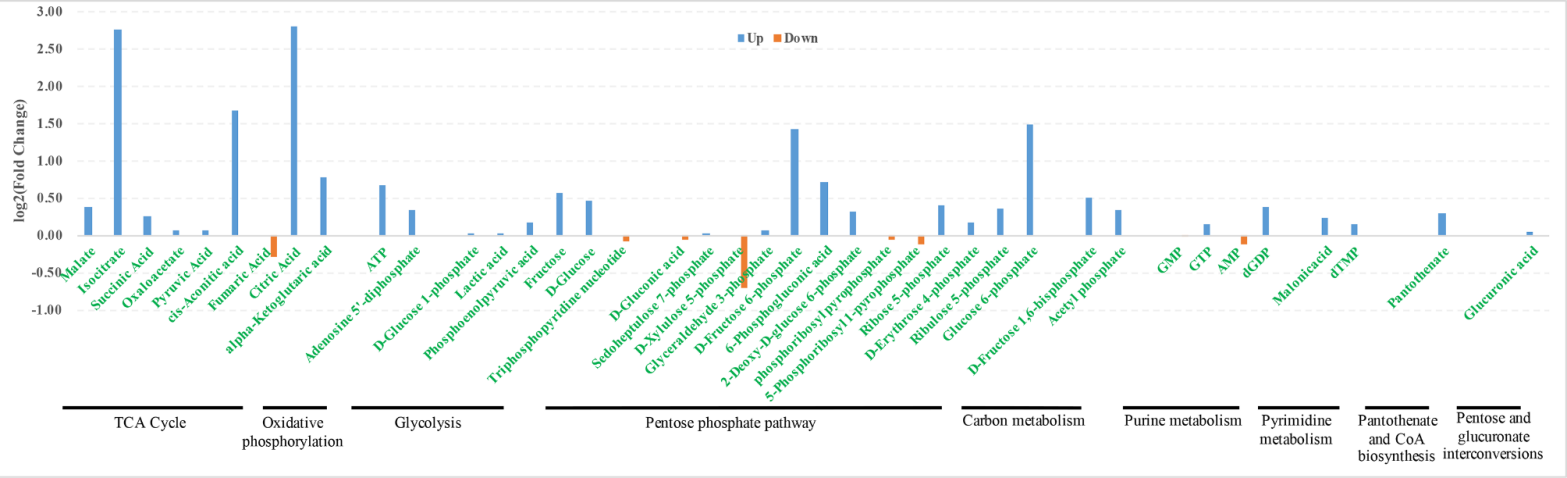
Relative quantitative analysis of central carbon-related substances (A. vs. P groups). The x‑axis represents central carbon-related substances and the y‑axis represents log2(fold change) values.

Upon Ats-1 expression, five proteins were up-expressed in the mitochondrial respiratory chain suggesting a potential influence on cellular energy metabolism. The central carbon pathway is the main source of energy for host cells. Consequently, we detected the expression levels of metabolites related to central carbon pathways after Ats-1 expression using targeted metabonomics. A total of 40 differential metabolites were subsequently detected (Figs. 6 and 7), with most differential metabolites involved in the pentose phosphate pathway. Thirteen of the metabolites (32.5%) exhibited altered expression, with nine up-regulated and four down-regulated (Fig. 7). Nine of these (22.5%) were involved in the TCA cycle pathway, of which eight were up-regulated and one was down-regulated. The metabolic pathway with the third largest number of differentially expressed metabolites was glycolysis, wherein the expression of six metabolites was altered (15%, five up-regulated and one down-regulated). Although the pentose phosphate pathway exhibited the largest number of differentially expressed metabolites, the pathway does not lead to the production or consumption of ATP. In contrast, the TCA cycle is an effective pathway to oxidize polysaccharides and other substances to conserve energy. Glycolysis is the pathway by which most organisms oxidize glucose, contribute to energy conservations, and involves many different metabolites. These data reveal that Ats-1 expression leads to the up-expression of metabolites within various energy conservation pathways and a general enhancement of cellular energy conservation.

### Verification of mitochondrial mRNA levels related to differential proteins based on qRT-PCR

**Fig. 8:**
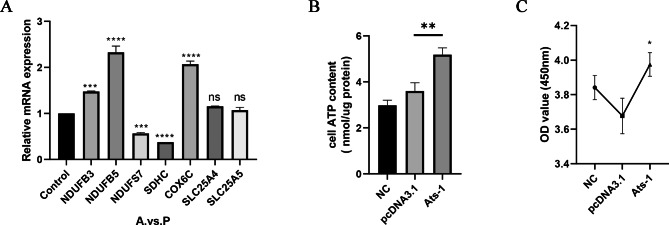
Ats-1 enhanced the respiratory activity and proliferation of HEK293T cells. (A) Analysis of qRT-PCR results. NDUFB5, COX6C, SLC25A4, and SLC25A5 were up-regulated after transfection with Ats-1 at the mRNA level, while SDHC and NDUFS7 were down-regulated. (B) ATP measurements in HEK293T cells. ATP levels significantly increased in cells after transfection with Ats-1 compared to transfection with an empty vector. (C) Cellular proliferation tests. Based on the CCK-8 method, the OD values were positively correlated with the number of living cells, thereby indirectly reflecting the proliferation of cells. Cellular proliferation was enhanced after transfection with Ats-1 compared to transfection with an empty vector. The results represent the results from three independent experiments. Data are shown as means ± SD from three independent tests. Ns: no significant difference, *: *p* < 0.05, **: *p* < 0.01, ***: *p* < 0.001, ****: *p* < 0.0001.

To evaluate the effects of Ats-1 expression on the mRNA expression of seven mitochondrial-related differentially expressed proteins, qRT-PCR assays were conducted. Compared with group P, Ats-1 expression significantly increased the mRNA expression of NDUFB3, NDUFB5, and COX6C and significantly inhibited the mRNA expression of SDHC and NDUFS7, but did not significantly affect the mRNA expression of SLC25A4 and SLC25A5 (Fig. 8 A). Comparative analysis of the qRT-PCR (Fig. 8 A) and PRM (Fig. 5) results demonstrated that the mRNA levels of NDUFB3, NDUFB5, COX6C, and SLC25A4 were consistent with protein levels (i.e., they were up-regulated), while the other three proteins exhibited differing trends. The combined results of iTRAQ, PRM, and qRT-PCR analyses revealed that the expression of Ats-1 affects the expression of NDUFB3, NDUFB5, NDUFS7, COX6C, and SLC25A5 within the respiratory chain of mitochondria (Table 2).

**Table 2:** Expression trends for seven proteins summarized in iTRAQ, PRM, and qRT-PCR.

Protein	iTRAQ	PRM	qRT-PCR
SLC25A5	up-regulated	up-regulated	unchanged
SLC25A4	up-regulated	down-regulated	unchanged
NDUFB5	up-regulated	up-regulated	up-regulated
NDUFS7	up-regulated	up-regulated	down-regulated
COX6C	up-regulated	up-regulated	up-regulated
NDUFB3	up-regulated	up-regulated	up-regulated
SDHC	up-regulated	down-regulated	down-regulated

### Ats-1 Increases ATP synthesis in HEK293T cells

The NDUFB3, NDUFB5, and COX6C proteins were up-regulated in the iTRAQ, PRM, and qRT-PCR analysis results, and they are all important components of the mitochondrial respiratory chain complex. Further, many metabolites in the TCA cycle were up-regulated, suggesting that the synthesis of ATP in host cells was altered after Ats-1 expression. Consequently, the levels of ATP synthesis in group A and group P were analyzed. The amount of ATP synthesis in group A was significantly higher than in group P (*p* < 0.01) (Fig. 8 B). Thus, Ats-1 expression affects the energy metabolism of host cells and increases ATP synthesis.

### Ats-1 Promotes the proliferation of HEK293T cells

Given that host cell ATP synthesis increases after Ats-1 expression, we speculated that Ats-1 expression promotes cellular proliferation. Detection of CCK8 was evaluated, revealing that the OD value of group A was significantly higher than that of group P (*p* < 0.05) (Fig. 8 C). Thus, the expression of Ats-1 promoted host cell proliferation.

### Ats-1 Inhibits apoptosis of HEK293T cells

**Fig. 9:**
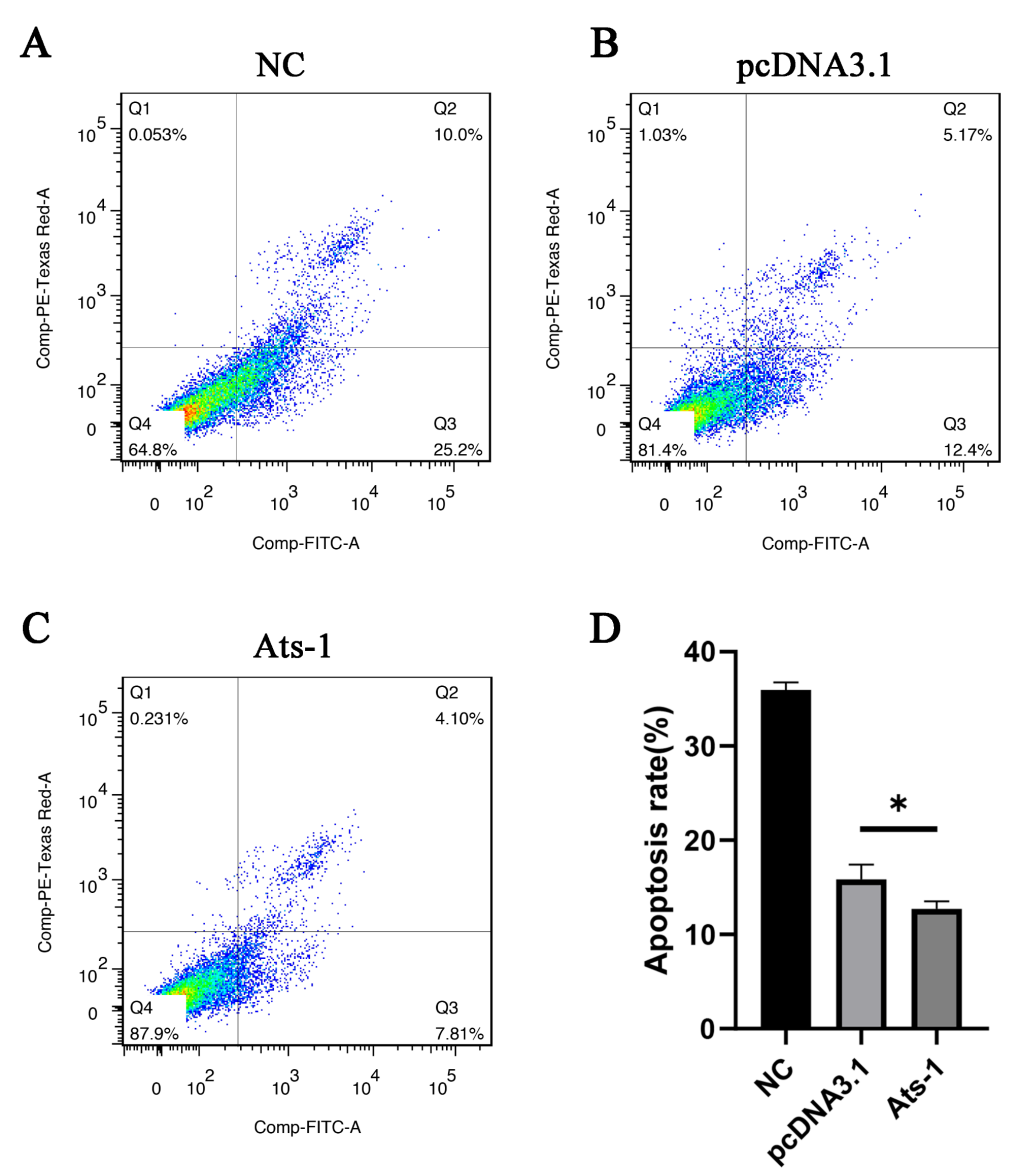
HEK293T cells apoptosis rate measurements. (A) Flow cytometry analysis of HEK293T cells apoptosis after 48 h of untransfection. (B) Flow cytometry analysis of HEK293T cells apoptosis 48 h after transfection with an empty vector. (C) Flow cytometry analysis of HEK293T cells apoptosis 48 h after transfection with Ats-1. (D) Statistical analysis of HEK293T cells apoptosis rate. Data are shown as means ± SD for three independent tests. *: *p* < 0.05.

The results begged the question of whether Ats-1 expression could alter apoptosis rates. The apoptosis rates of each treatment group were consequently evaluated by flow cytometry. The apoptosis rate was 35.2% in group H (Fig. 9 A), 17.57% in group P (Fig. 9 B), and 11.91% in group A (Fig. 9 C). The apoptosis rate in group A was significantly lower than in group P (*p* < 0.05) (Fig. 9 D). Thus, Ats-1 expression can inhibit the apoptosis of HEK293T cells.

## Discussion

Bioinformatics analysis helps us to better understand Ats-1. Online software predicted the presence of the Sec signal peptide and Tat signal peptide in Ats-1 (Supplementary Fig. 1 A), which are associated with protein secretion. Combined with GO functional annotation and KEGG enrichment analysis of the A .vs. P groups, the differential proteins were enriched in both the membrane and membrane components in the cellular component category (Fig. 3), and enriched in protein output in the KEGG pathway (Fig. 4). Ats-1 may regulate the process of protein secretion in host cells. Ats-1 also has a mitochondrial transfer signal, which is consistent with the results that Ats-1 targets translocated mitochondria [12]. In the prediction results, Ats-1 had no nuclear localization signal or nuclear output signal. There is currently no relevant literature on Ats-1 entry into the nucleus. Because the team has not yet isolated *A. phagocytophilum* and cannot directly infect host cells (HL-60) with pathogens, and because HEK293T cells are easily transfected with recombinant plasmids, this study observes Ats-1 transfection into HEK293T cells.

The iTRAQ and PRM (Fig. 5) results did not perfectly coincide and this difference may be due to several factors. First, the instruments and quantitative principles behind the two technologies differ. Moreover, iTRAQ methods are not targeted and quantitative, but rather are a high-throughput identification method for proteins. Consequently, some false positive results can be achieved. Thus, PRM and qRT-PCR are needed to verify iTRAQ results. Five of the seven target proteins (NDUFB3, NDUFB5, NDUFS7, COX6C, and SLC25A5) exhibited the same expression trend (i.e., up-regulation) that nevertheless confirm the repeatability and reliability of iTRAQ data. NDUFS7 mRNA levels were down-regulated (Fig. 8 A), while protein levels were up-regulated (Fig. 5). Previous studies have shown that post-transcriptional regulation, translation regulation, and degradation regulation play important roles in determining protein concentrations relative to mRNA levels [27]. Therefore, corresponding correlations between protein levels and mRNA levels may not always coincide. Nevertheless, protein functionality can still be assessed and the PRM results at the protein level were the most significant aspects of this study.

**Fig. 10:**
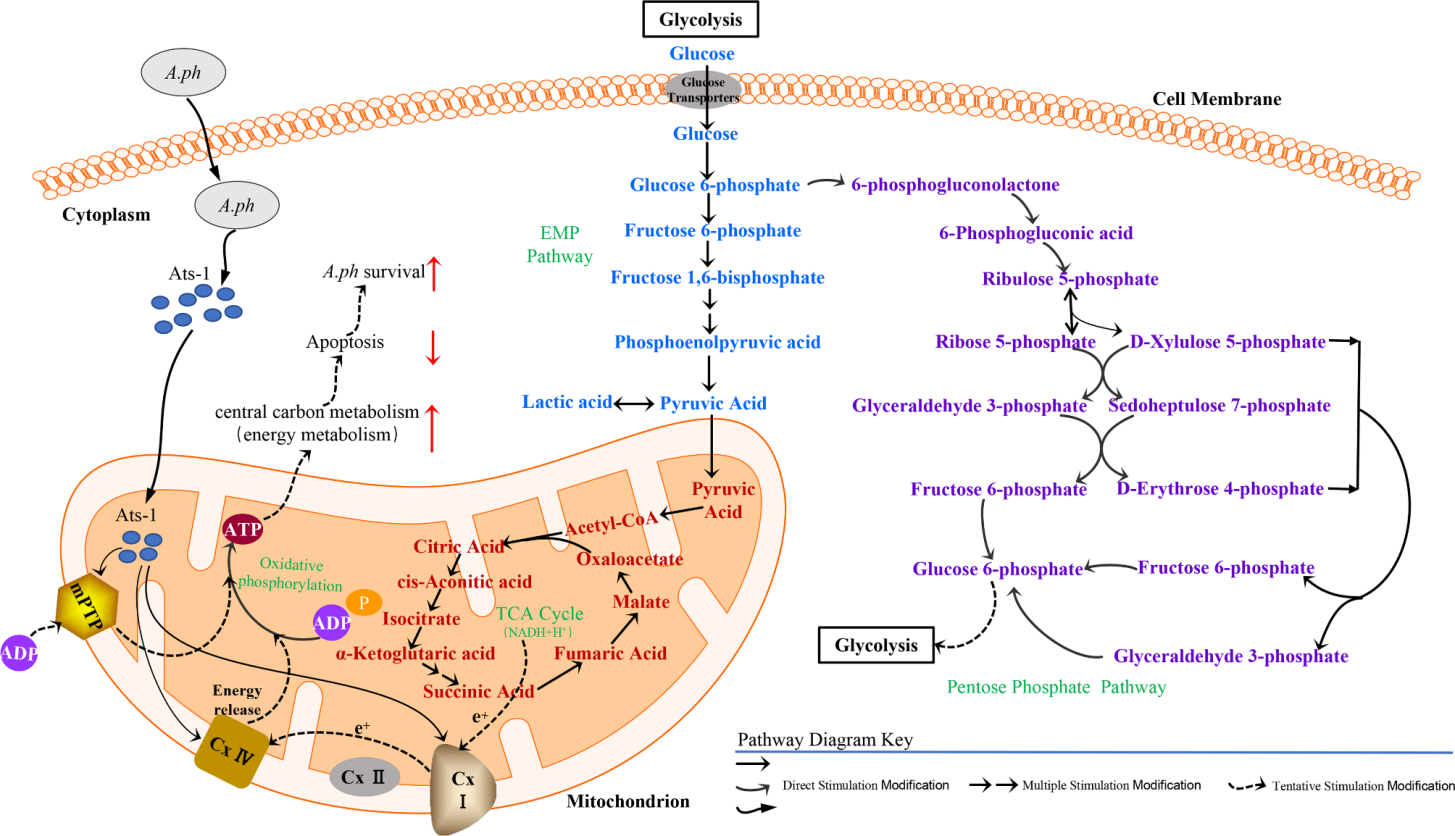
Schematic showing the role of Ats-1 in host cells. The solid lines represent known results and the dashed lines represents hypothesized activities.

Based on the results of this study, the regulation of Ats-1 in host cells was hypothesized (Fig. 10) to clarify the roles and functional mechanisms of Ats-1. After *A. phagocytophilum* enters the host cell, it secretes Ats-1 protein within the cytoplasm and a part of the Ats-1 protein enters the mitochondria, leading to direct up-regulation of complex I and complex IV components (NDUFB3, NDUFB5, NDUFS7, and COX6C), thereby enhancing the function of complexes I and IV. The expression of Ats-1 in cells can directly or indirectly enhance the expression intensity of the pentose phosphate, glycolysis, and TCA cycle pathways [25, 26] within mitochondria. Numerous redox reactions then take place in the glycolysis, tricarboxylic acid cycle, and pentose phosphate pathways [25, 26], resulting in increased production of hydrogen protons (H^+^) and electrons (e^−^). According to Mitchell’s [28] chemical permeation theory, hydrogen protons (H ^+^) and electrons (e ^−^) transfer in the mitochondrial electron transport chain and couple with ATP synthesis via the oxidative phosphorylation coupling reaction, resulting in increased ATP production. These processes inhibit the apoptosis of host cells, promote the proliferation of host cells, and thereby contribute to the survival of *A. phagocytophilum* in host cells. Under normal circumstances, mPTP allows some small ions to pass freely through the membrane and activate ATP synthetase through oxidative phosphorylation to maintain mitochondrial membrane potential and ion balance inside and outside the cell. The component protein (SLC25A5) of mPTP is up-regulated in this pathway and the biological function is enhanced, thereby leading to increased ATP synthetase activity and increased ATP production. Both of the two regulation routes can increase metabolic energy and inhibit host cell apoptosis, thereby providing more time for *A. phagocytophilum* replication in host cells.

Numerous bacterial proteins have been shown to inhibit host cell apoptosis via mitochondrial action. For example, the outer membrane protein PorB of *Neisseria meningitides* [29] and the *Chlamydia*-secreted protease-like activity factor termed CPAF [30] can regulate host cell apoptosis through various mitochondrial mechanisms. Niu et al. [12]showed that Ats-1 blocks the loss of mitochondrial membrane potential and inhibits host cell apoptosis induced by etoposide. This study also shows that Ats-1 can inhibit host cell apoptosis through mitochondrial activities. In contrast to previous studies, Ats-1 expression inhibited host cell apoptosis by up-regulating five proteins (NDUFB3, NDUFB5, NDUFS7, COX6C, and SLC25A5) within the mitochondrial respiratory chain. It is unclear whether these five upregulated proteins (NDUFB3, NDUFB5, NDUFS7, COX6C, and SLC25A5) act on other signaling pathways to regulate cellular pathways. Studies in recent years have shown that NDUFB3 is a key protein involved in the oxidation of phosphate and is involved in Parkinson’s disease, Huntington’s disease, and Alzheimer’s disease pathways [31]. Further, some researchers have speculated that COX6C is an important component of the apoptotic pathway and may play a regulatory role in the early stages of influenza infection [32]. Whether the five up-regulated proteins identified here play an important role in *A. phagocytophilum* infection nevertheless requires further study.

*A. phagocytophilum* Ats-1 and *E. chaffeensis* Etf-1 are both T4SS effectors that share 21% homology at the amino acid level [33]. Etf-1 is located in mitochondria and blocks the apoptosis of host cells that is mediated by mitochondria, such that infected host cells can survive and *E. chaffeensis* can replicate in cells [33]. *A. phagocytophilum* Ats-1 also targets translocation to the host cell mitochondria to inhibit host cell apoptosis, thereby promoting the survival of *A. phagocytophilum* in host cells [12]. Thus, the functions of the two proteins are similar. Intracellular Etf-1 nano-antibody (Nbs) is a feasible treatment for blocking intracellular *E. chaffeensis* infections [33]. Consequently, the development of intracellular anti-Ats-1-Nbs would be an attractive area to research.

## Conclusion

In this study, *A. phagocytophilum* Ats-1 was observed to inhibit host cell apoptosis by up-regulating proteins involved in respiratory chains and mPTP within host cell mitochondria, providing new insights regarding the intracellular parasitism mechanism of *A. phagocytophilum*. A total of 407 up-regulated proteins and 455 down-regulated proteins were identified by iTRAQ analysis in HEK293T cells expressed by Ats-1. The function and classification of these differentially expressed proteins were annotated by GO and KEGG databases. In addition, seven differentially expressed proteins related to mitochondrial respiratory chain function were verified by PRM and qRT-PCR analyses. The expression of Ats-1 upregulated five proteins (NDUFB3, NDUFB5, NDUFS7, COX6C, and SLC25A5) involved in the mitochondrial respiratory chain and that led to increased ATP synthesis, enhanced cellular proliferation, and decreased apoptosis rates, ultimately providing more time for *A. phagocytophilum* to replicate and survive in host cells.

## Electronic supplementary material

Below is the link to the electronic supplementary material.

Supplementary Material 1

## Data Availability

The mass spectrometry proteomics data have been deposited to the ProteomeXchange Consortium (http://proteomecentral.proteomexchange.org) via the iProX partner repository [34] with the dataset identifier PXD029412. ‍Other data generated or analyzed during this study are included in this published article.
