# Supplementary material for: Multiomics analyses reveals Anaplasma phagocytophilum Ats-1 induces anti-apoptosis and energy metabolism by upregulating the respiratory chain-mPTP axis in eukaryotic mitochondria
Source: BMC Microbiol. 2022 Nov 11;22:271. doi: 10.1186/s12866-022-02668-x (PMC9650841; doi:10.1186/s12866-022-02668-x)
Supplement: Supplementary file 1 — Supplementary Material 1 [file 12866_2022_2668_MOESM1_ESM.pdf]

Supplementary material

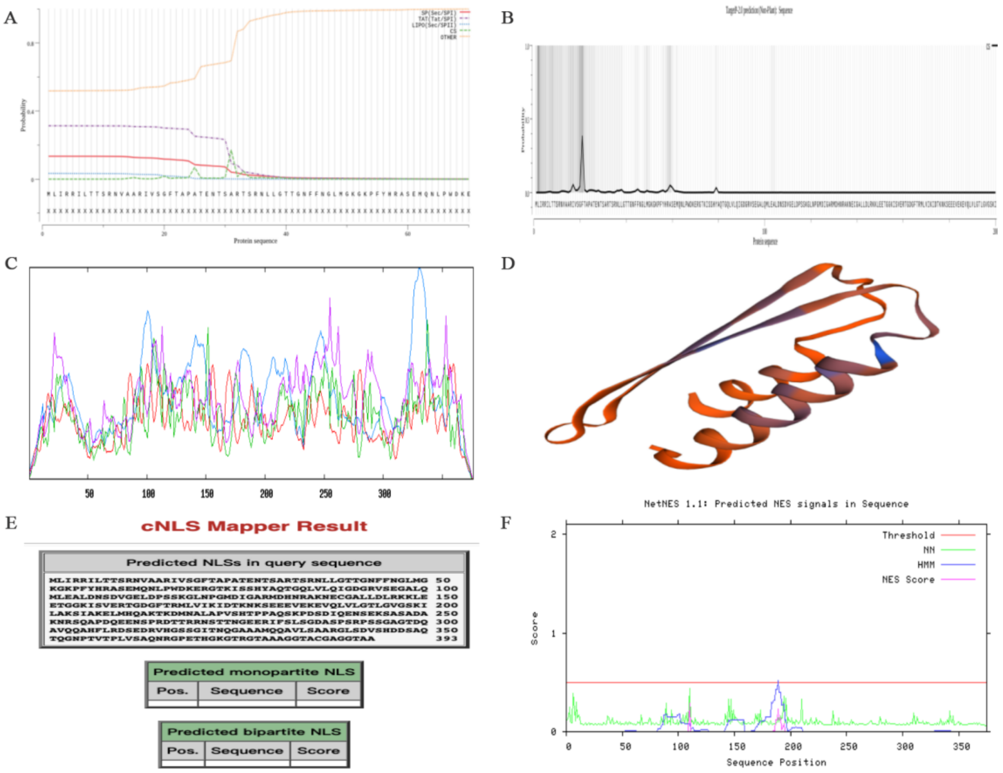

Supplementary Figure 1. (A) Ats-1 signal peptide prediction. (B) Ats-1 protein mitochondrial transfer signal peptide prediction. (C) Ats-1 protein secondary structure prediction. (D) Tertiary structure prediction of Ats-1 proteins. (E) Ats-1 protein nuclear localization signal (NLS) prediction. (F) Ats-1 protein nuclear export signal (NES) prediction.

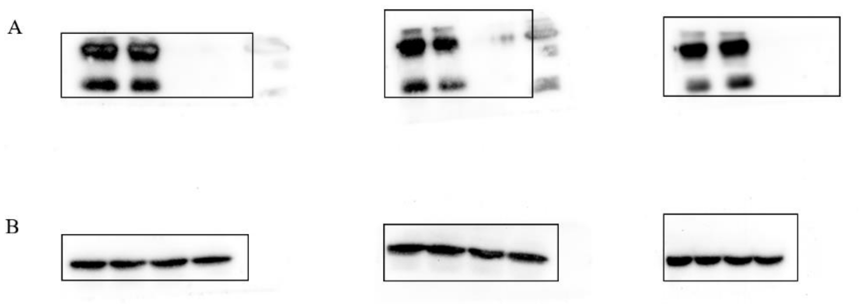

Supplementary Figure 2. Original diagram of Fig. 1A. Blots were hybridized to antibodies after a cut. (A) Original blot for Ats-1 protein with three replicates. (B) Profiles of blots for β-actin protein with three replicates.

Supplementary Table 1. qPT-PCR primers used in this study

Primers Name	Sequences(5′to 3′)	TM	Accession
NDUFB3	F: TTGGAGATACATGGGTGGCTTTGC	59.9	NC_000002
	R:GCTACCACAAATGCAGCAAATCCC	59.5	
NDUFB5	F: TGAGAGGAGATGGACCCTGG	58.8	NC_000003
	R: TGTCAGGAGTTGCTTTCGGAG	57.4	
NDUFS7	F: CATCGTGCCCGTGGACATCTAC	60.9	NC_000019
	R: TCCGCTCCCGCTTGATCTTCC	62.8	
COX6C	F: AAGTTTTGCCAAAACCTCGGATGC	58.8	NC_000008
	R: CCCCAGGGATAGCACGAATGC	61.5	
SLC25A5	F: TTCGCCGCCGCATGATGATG	61.9	NC_000023
	R: GAGCAATCTTCCGCCAGCAGTC	61.6	
SLC25A4	F: TGTGGATCGGCATAAGCAGTTCTG	59.5	NC_000004
	R: GGGTAGACAAAGCAAAGGGAGGTG	60.4	
SDHC	F: CATTGCCTCCGAGCCCACTTTAG	60.9	NC_000001
	R: TGGGGAGACAGAGGACGGTTTG	61.9	
GAPDH	F: GGAGCGAGATCCCTCCAAAAT	61.6	NC_000012
	R: GGCTGTTGTCATACTTCTCATGG	60.9	
